# Age, anti-müllerian hormone, antral follicles count to predict amenorrhea or oligomenorrhea after chemotherapy with cyclophosphamide

**DOI:** 10.1186/s13048-015-0209-4

**Published:** 2015-12-14

**Authors:** Ângela Marcon D’Avila, Vanderlei Biolchi, Edison Capp, Helena von Eye Corleta

**Affiliations:** Graduate Program in Medicine: Medical Sciences, Universidade Federal do Rio Grande do Sul School of Medicine, Bento Gonçalves, RS Brazil; Graduate Program in Biological Sciences: Physiology, Instituto de Ciências Básicas da Saúde, Universidade Federal do Rio Grande do Sul, Lajeado, RS Brazil; Department of Obstetrics and Gynecology, Hospital de Clínicas de Porto Alegre, Sul School of Medicine, Universidade Federal do Rio Grande do Sul, Porto Alegre, RS Brazil; Department of Gynecological Endocrinology and Reproductive Medicine, University Hospital Heidelberg, Heidelberg, Germany; GENERAR, Porto Alegre, RS Brazil; Serviço de Ginecologia e Obstetrícia - Hospital de Clínicas de Porto Alegre Rua Ramiro Barcelos, 2350/11° andar, Porto Alegre, CEP 90035-903 RS Brazil

**Keywords:** Anti-Müllerian hormone, Antral follicles count, Ovarian reserve, Amenorrhea related chemotherapy, Anovulation related chemotherapy

## Abstract

**Background:**

A cohort study was performed to identify ovarian reserve markers (ORM) that predicts amenorrhea or oligomenorrhea 6 months after cyclophosphamide CTX in women with breast cancer.

**Methods:**

52 eumenorrheic patients with breast cancer were enrolled. FSH, anti-Müllerian hormone (AMH), antral follicles count (AFC) were measured before and 6 months after CTX. A logistic regression for independent samples and determination of the ROC curve were performed.

**Results:**

The age of 32 years presented 96 % of sensitivity and 39 % of specificity to predict amenorrhea or oligomenorrhea with ROC area under the curve (AUC) of 0.77. ovarian reserve marker (ORM) with power to predict amenorrhea or oligomenorrhea in women after CTX were AMH <3.32 ng/mL (sensitivity of 85 %, specificity of 75 % and AUC 0.87), AFC <13 follicles (sensitivity 81 %, specificity 62 %, AUC 0.81). AMH cutoff to predict amenorrhea was 1.87 ng/mL (sensitivity 82 %, specificity 83 %, AUC 0.84) and AFC cutoff was 9 follicles (sensitivity 71 %, specificity 78 %, AUC 0.73).

**Conclusions:**

≥32-years-old women, AMH <3.32 ng/mL and AFC <13 follicles determined significantly higher risk of amenorrhea or oligomenorrhea after CTX with cyclophosphamide. The ORM age (≥32 years) analyzed together with AMH or AFC increases sensitivity and specificity in predicting amenorrhea or oligomenorrhea.

## Background

Currently, around 5 % of the malignant neoplasias affect people younger than 35 years old [1]. In the United States, approximately 50,000 new cases of malignancy are diagnosed per year in young people [2]. Breast cancer is the second most frequent malignancy, affecting around 11,000 women per year [3]. The prognosis of malignancy improved because of early diagnosis and CTX treatment, which, despite their effectiveness against the disease, can have damaging effect on the gonadal function, compromising reproductive future [2, 4].

Among chemotherapic agents, the alkylating are those with the higher gonadotoxic potential. The impact of chemotherapy on the reproductive future of the patients exposed to CTX is determined by their age, type and dose of chemotherapic agents, length of treatment and chemotherapic agents association [5–7]. However, the literature is poor about the importance of assessing ovarian reserve (OVR) before undergoing CTX treatment to predict the reproductive prognosis after the cure of the disease [4, 8].

OVR refers to quantity and, to some authors, quality of follicles present in ovaries at a given time. It is the measure of oocyte production and consequently reproductive potential [9, 10]. Its evaluation is through serum analysis of FSH, estradiol, inhibin and anti-Müllerian hormone (AMH) and through ultrasonographic counting of antral follicles (AFC) [11]. AFC refers to the number of follicles visible in ovaries during ultrasound scan in the first days of the menstrual cycle. There is controversy in the literature about the size of follicles that must be counted, 2 to 5 mm or 2 to 10 mm [1, 9]. AMH is a dimeric glycoprotein that determines the anatomy of the female internal genitalia and works in the development of primordial and growing follicles. It has been hypothesized as the most reliable ovarian reserve marker (ORM), because its levels do not vary during the menstrual cycle and it is not detectable in menopause [1, 12].

As fertility preservation may be a priority for young women with cancer, we analyzed which ORM can be used as predictors of anovulation (oligomenorrhea or amenorrhea) 6 months after CTX with cyclophosphamide in women with breast cancer.

## Results and discussion

The mean age of patients was 35.3 ± 3.8 years (range 27–40 years) and the age distribution is presented in Table 1. The main histological type of breast cancer was invasive ductal carcinoma (98 %). Forty percent of patients underwent breast-conserving surgery prior to or followed by CTX treatments and 75 % of patients underwent adjuvant radiotherapy. The follow-up mean time was 14 ± 3 months since the first assessment. Five patients quit the follow-up study. Three for recurrence of the disease, making it impossible to attend the assessment appointment, one gave up the treatment and another died. Mean cycles number for ciclofosfamide was 4.5 per patient and 1,4 for paclitaxel (34 patients used paclitaxel associated to ciclofosfamide). There was no difference in the number of cycles between patients with regular cycles and those with amenorrhea and oligomenorrhea.

**Table 1:** Clinical characteristics of patients

	n (total = 52)	%
Age (years)		
<32	8	15
≥32	44	85
32–35	17	33
36–38	11	21
39–40	16	31
BMI (kg/m^2^)*		
Eumenorrheic	26.16 ± 16.0	
Anovulatory	24.4 ± 3.3	
Tumor type		
Ductal invasive	51	98
Paget Disease	1	2
Chemotherapy		
Neoadjuvant	21	40
Adjuvant	31	60
Radiation therapy	41	79

*P = 0.18

Patients with 32 years of age or younger had levels of AMH similar to those of patients older than 32 years old (5.41 [0.20–24.55]) and 2.32 [0.0–13.66], respectively) and AFC were significantly higher than patients older than 32-years (respectively 14.0 [8.0–20.0] and 10.0 [6.0–18.0], *p* = 0.061 and *p* = 0.023). FSH was not different in this analysis.

Thirty-nine percent of patients were amenorrheic 6 months after CTX, 21 % were oligomenorrheic and 40 % eumenorrheic. The baseline ORM were related to menstrual outcome after CTX, showing that the amenorrheic women and women with regular menstrual cycles are statistically different with regard to age, AMH and AFC, with *p* = 0.006; <0.001; 0.003, respectively. Comparing oligomenorrheic patients (irregular menstrual cycles) versus amenorrheic and oligomenorrheic versus eumenorrheic patients no differences were found in age, AMH or AFC (Table 2).

**Table 2:** ORM before CTX and menstrual outcome 6 months after CTX

	Eumenorrhea	Oligomenorrhea	Amenorrhea
	(19 patients)	(10 patients)	(18 patients)
Age (years)	32.9 ± 3.5	35.7 ± 3	36.9 ± 3.4*
AMH (ng/mL)	5.34 (2.71–8.15)	3.21 (1.55 – 4.74)	0.92 (0.24 – 1.66)**
AFC (follicles)	13.5 (11–16)	10 (8–12)	9 (7.5–12)***

Eumenorrhea X Amenorrhea *p = 0.006; **p < 0.001; ***p = 0.003

Aiming to define the risk predictors for significant OVR loss of after CTX, the researchers decided to create a group with menstrual irregularity (oligomenorrheic and amenorrheic patients) in order to compare it with the group that remain eumenorrheic after CTX. The mean age of women with oligomenorrhea or amenorrhea six months after CTX was 36.5 ± 3.8 years, while women with regular cycles had a mean age of 32.9 ± 3.5 years (*p* = 0.02). The AFC, independently of age, was statistically significant for risk prediction for oligomenorrhea or amenorrhea (*p* = 0.001 and confidence interval [CI] 0.548–0.931). AMH also showed to be a good predictor for the decline of reproductive function, with *p* < 0.001 and CI 0.541–0.941 (Table 3).

**Table 3:** Baseline ORM related to menstrual outcome 6 months after CTX group of anovulatory patients (oligomenorrheic and amenorreic) and group of ovulatory patients (eumenorrheic)

	Eumenorrhea (*n* = 19)	Oligomenorrhea + Amenorrhea (*n* = 28)
Age (years)	32.9 ± 3.5	36.5 ± 3.8*
AMH (ng/mL)	5.34 (2.71–8.15)	1.31 (0.72 – 2.89)**
AFC (follicles)	13.5 (11–16)	9 (7.75–12)**

Oligomenorrhea + Amenorrhea X Eumenorrhea (Regular cycles): *p = 0.02; **p < 0.001

Baseline estradiol levels, smoking habits and body mass index were not different between the oligomenorrheic and amenorrheic group and the regular cycling group 6 months after CTX.

**Fig. 1:**
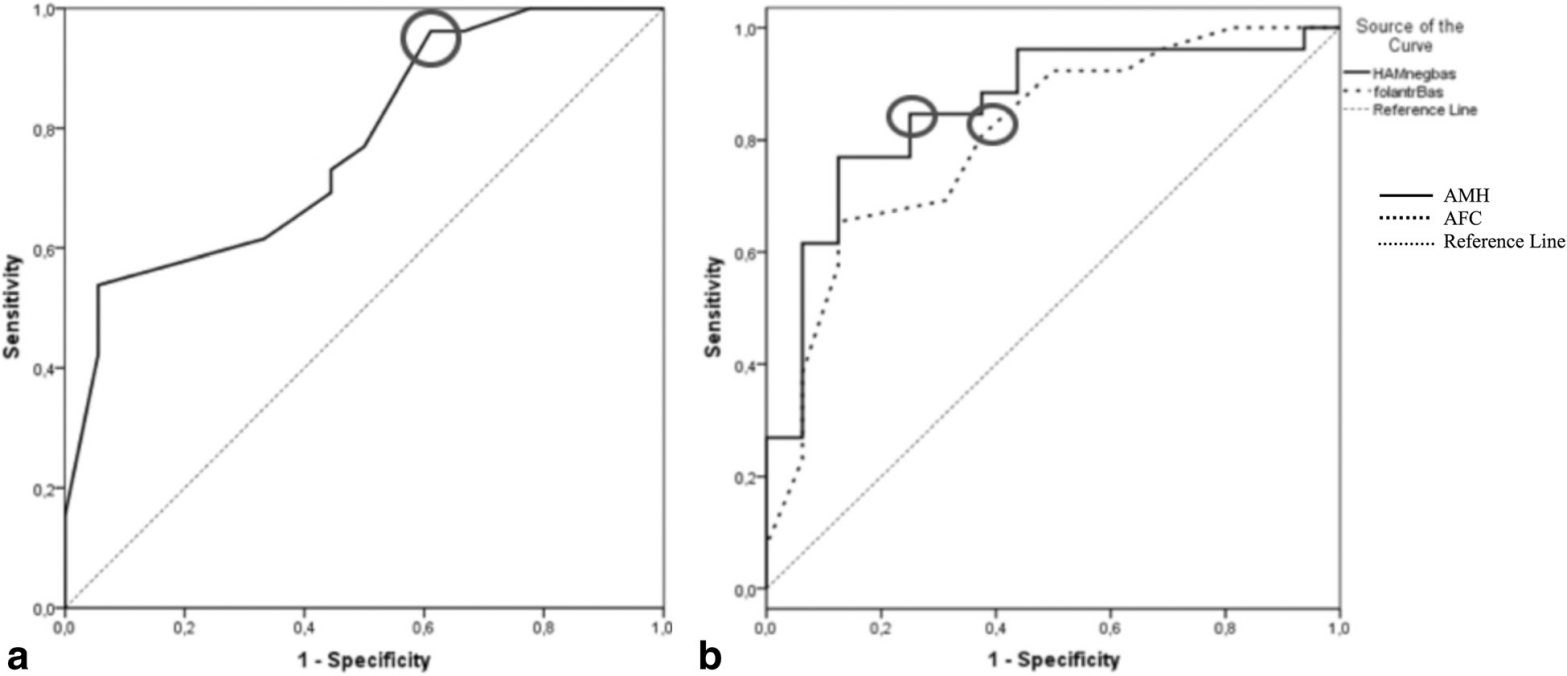
**a** ROC curve for age in predicting amenorrhea or oligomenorrhea 6 months after CTX. AUC = 0.77; circle = age > 32. **b** AMH and AFC ROC curve for amenorrhea or oligomenorrhea 6 months after CTX. Circles on the curves indicate the cut off with better sensitivity and specificity in predicting cycle irregularity. AUC (AMH) = 0.86. AUC (AFC) = 0.81

Table 4 shows ORM analyzed through logistic regression. The multiple regression used to analyze many variables together was not possible because of the multicollinearity of age, AMH and AFC, therefore the analysis was independently performed for each variable controlled by age. The age of 32 showed sensitivity 96 % and specificity 39 % to predict cycle irregularity. The odds ratio (OR) for oligomenorrhea or amenorrhea in patients ≥ 32-years-old was 15.9 with CI 1–145, *p* = 0.03 (Table 4). The Positive Predictive Value (PPV) of ≥ 32 years for anovulation was 70 % and the Negative Predictive Value (NPV) was 87 %. Figure 1 represents the ROC curve that defines the most accurate age to predict oligomenorrhea or amenorrhea, 32 years, with AUC 0.77 (Fig. 1). Analyzing exclusively amenorrheic women 6 months after CTX, the age of 35 years or more predicted amenorrhea with sensitivity 76 %, specificity 66 %, CI 0.53 – 0.85 and AUC of 0.69.

**Table 4:** ORM cut off in predicting amenorrhea or oligomenorrhea 6 months after CTX with cyclophosphamide

	Cut Off	p	OR	CI	Sensitivity	Specificity
Age	≥32 anos	0.03	15.9	1–145	96 %	39 %
AMH	<3.32 ng/mL	0.04	10.3	2.14–50.37	85 %	75 %
AFC	<13 folículos	0.059	4.43	0.943–20.8	81 %	62 %
FSH (≥32 years)	≥6.66 UI/mL	0.021	6.36	1.11–36.41	60 %	82 %

FSH was not a predictor of oligomenorrhea and amenorrhea after CTX. However, women with ≥ 32-years-old and FSH ≥ 6.66 UI/mL had significantly higher oligo or amenorrheic cycles than women with FSH lower than this cut off, with OR 6.36 (CI 1.11–36.41), *p* = 0.021 with sensitivity 60 % and specificity 82 % (Table 4). The PPV of FSH 6.66 IU in women older than 32 years was 73 %, while the NPV was 55 %.

The cut off for AFC was <13 follicles to predict oligomenorrhea or amenorrhea. Patients with <13 follicles presented higher risk of oligomenorrhea or amenorrhea 6 months after CTX, with sensitivity 81 %, specificity 62 % and AUC 0.81 (Fig. 1b). The OR was 4.43 (CI 0.943–20.8, *p* = 0.059) (Table 4). The PPV of AFC of <13 follicles for oligomenorrhea or amenorrhea was 78 %, while the NPV was 67 %. Although there was no statistical significance, 33 % of the patients with AFC ≥ 13 developed oligomenorrhea or amenorrhea, while 78 % with AFC <13 developed the same outcome.

Values of AMH under 3.32 ng/mL presented sensitivity 85 % and specificity 75 % to occurrence of oligomenorrhea or amenorrhea. The ROC AUC for 3.32 ng/mL was 0.86 (Fig. 1b). Only 20 % of the patients with AMH > 3.32 ng/mL presented oligomenorrhea or amenorrhea, while 80 % of the women with AMH under this cut off resulted in the same outcome. OR for oligomenorrhea or amenorrhea in women with AMH levels < 3.32 ng/mL was 10.3 (CI 2.14 – 50.37, *p* = 0.04) (Table 4). The PPV of AMH of 3.32 ng/mL oligomenorrhea or amenorrhea was 79 %, while the NPV was 75 %.

Analyzing exclusively amenorrheic women 6 months after CTX, the cut off to predict amenorrhea was 1.87 ng/mL for AMH, sensitivity of 82 %, specificity of 83 %, AUC of 0.84) and 9 follicles for AFC, sensitivity 71 %, specificity 78 % and AUC 0.73.

Table 5 shows the analysis in series and in parallel of the AMH (<3.32 ng/mL) and AFC (<13) with the clinical ORM age ≥ 32 years aimed to increase sensitivity in predicting oligomenorrhea or amenorrhea when tests were used in parallel and to increase specifity when used in series. The specifity for oligomenorrhea or amenorrhea when age (≥32 years) was analyzed in series with AMH (<3.32 ng/mL) was 80 % and with AFC (<13) was 78 %. The analysis of ORM was not influenced by the number of cycles (4 or 6), dose of CTX calculated according to body mass index, or by radiotherapy association. The cyclophosphamide dosage was 600 mg/m2, 43 patients did 4 cycles and 6 patients did 6 cycles. The menstrual pattern and the ovarian reserve markers were not different between the groups. The AMH was 0.57 ± 2.29 and 0.16 ± 1.01 and AFC was 5.2 ± 3.1 and 6.0 ± 3.3 in the groups of 4 and 6 cycles respectively. The Pearson correlation between cyclophosphamide dose and AMH, FSH and AFC 6 months after CTX was, respectively, 0.24 with *p* = 0.944, − 0.442 with *p* = 0.174 and 0.21 with *p* = 0.342.

**Table 5:** AMH cut off (3.32 ng/mL) analyzed concomitantly with other ORM in parallel and in series, the prediction of amenorrhea or oligomenorrhea

Age ≥ 32 years	Parallel test		Series test	
	Sensivity	Specificity	Sensivity	Specificity
AMH ≤3,32	99,4 %	26 %	81 %	80 %
AFC <13 follicles	99 %	24 %	78 %	78 %

This cohort study evaluated the sensitivity and specificity of the ORM (age, FSH, AMH, AFC) and correlate them with the occurrence of oligomenorrhea or amenorrhea in young women with breast cancer exposed to CTX with cyclophosphamide (there is one study in women younger than 40 years old) [13]. The occurrence of amenorrhea in these patients has been reported as up to 70 %, varying with the age of the woman [13]. In our study, the incidence of oligomenorrhea or amenorrhea, associated with decreased fertility, 6 months after CTX was 60 % and the incidence of amenorrhea (ovarian failure or premature menopause) in the same period was 39 %.

Age and CTX treatment are among the main predictor factors for occurrence of menopause in eumenorrheic women with breast cancer [14]. Levels of AMH before gonadotoxic CTX also seem to be a predictor [15]. Anderson et al. followed women with breast cancer for 5 years and demonstrated that AMH levels lower than 1.9 ng/mL are predictor of ovarian failure [16]. Dillon et al., recently described in 33 women treated with alkilating agents that pretreatment AMH level was associated with the rate of recovery of AMH after treatment. AMH level >2 ng/mL recovered at a rate of 11.9 % per month after chemotherapy, whereas participants with pretreatment AMH levels ≤2 ng/mL recovered at a rate of 2.6 % per month after therapy [14]. According to Van Roiij et al., patients with irregular cycles already show significant decline in their reproductive function [12]. Singh et al. reported the importance of predicting the decline of OVR in patients with breast cancer, even when there is no amenorrhea [17]. Thus, the outcome in our study was considered oligomenorrhea or amenorrhea, comparing it to regular cycles (ovulatory), women 6 months after CTX. It was the author’s decision to assess together the oligomenorrhea and amenorrhea group since it has been demonstrated that both represent a sign of lower fertility [18], related to anovulation. In the literature there are no studies that consider oligomenorrhea or amenorrhea as an outcome of CTX gonadotoxicity.

Petrek et al. found a greater incidence of amenorrhea after CTX in women older than 40 years of age [19]. Gracia et al., in a transversal study analyzing the ovarian reserve in 61 cancers survivors, compared with 67 healthy, similarly aged unexposed subjects; and 69 regularly menstruating women of late reproductive age, concluded ORM (FSH, AMH, and AFC) differed between exposed vs. unexposed subjects; and are similar with women of late reproductive age [4]. Tiong et al. described amenorrhea to be reversible 15 months after CTX [20]. Our study, however, enrolled 52 women under 40 years old and was able to define more precisely which women, younger than 40 years old, will have greater impairment in their reproductive capacity. Age that determines a higher incidence of amenorrhea was 35. However, to define at which age women will loss of the reproductive function, even with irregular cycle presence, we chose an age cut off with a higher sensitivity, without loss of specificity, in predicting risk of anovulation, i.e., 32 years. The age of ≥32 years shows sensitivity near 100 % for oligomenorrhea or amenorrhea and ORM (AMH and AFC) in this group were significantly lower than in younger women. Therefore, for patients without offspring, 32 years seems to be the age to alert doctors and patients to preserve fertility prior to CTX.

FSH was not a good ORM, as demonstrated by other authors. Significant FSH changes occur only with amenorrhea being a marker of ovarian failure and not of decline in OVR [7, 21]. However, FSH greater than or equal to 6.66 IU / mL in women over 32 years of age seems to carry a higher risk of cycle irregularity after CTX. Similar analysis was not found in the literature.

With respect to the AFC, it was demonstrated that patients witr less than 13 follicles, independent of age, are more susceptible to oligomenorrhea or amenorrhea (OR 4.43, sensitivity of 81 % and specificity of 62 % and *p* = 0,059). We believe that no statistical significance for the AFC in the prediction of oligomenorrhea or amenorrhea in this group of patients is due to the calculation of the sample size, which aimed to find differences in the AMH levels. Anderson et al., in a similar study, did not describe the cut off in the number of antral follicles associated with amenorrhea. They reported that the AFC mean before CTX in women who maintained ovulatory cycles was 19 versus 8 in those who developed amenorrhea [16].

AMH values greater than or equal to 3.32 ng/mL, regardless of the patient’s age, are protective for the occurrence of oligomenorrhea or amenorrhea after CTX with cyclophosphamide, with sensitivity 85 % and specificity 75 %. Even with a high confidence interval in this analysis, the OR was 10.3 meaning that patients with AMH < 3.32 ng/mL are 10 times more likely to develop oligomenorrhea or amenorrhea.

Previously, Anders et al. demonstrated that AMH values below 1.09 ng/mL increased the risk of amenorrhea after CTX in the evaluation of 44 patients with breast cancer and a mean age of 40 years [22]. Anderson et al. in 2011 conducted a similar study and found that value of AMH of 1.9 ng/mL with sensitivity of 54 % and specificity of 92 %, ROC AUC 0.91 and OR 7.0 as a predictor of risk for amenorrhea during a 5-year follow-up study of 42 women exposed to CTX [16]. Our results were similar to the occurrence of amenorrhea, finding AMH values below 1.87 ng/mL for this outcome. However, we prioritized the group of patients who would need counseling for fertility preservation, that is, those who would have their fertility compromised either by anovulation (amenorrhea and oligomenorrhea). Literature only shows studies that have evaluated OVR of patients who developed exclusive amenorrhea, patients with irreversible ovarian failure [16, 22]. Aiming to increase the sensitivity in predicting decrease fertility (anovulation) and provide the greatest safety in medical indication for fertility preservation techniques, this study accomplished association between ORM to increase power in predicting oligo or amenorrhea. The ORM with great sensibility to predict amenorrhea and oligomenorrhea was age ≥ 32 years, when this age was analyzed in parallel with AMH or AFC the sensibility reaches 99 %, and in series the specific was around 80 %.

## Conclusions

Thirty-two years old or older women, serum AMH < 3.32 ng/mL or AFC <13 offer a higher risk of amenorrhea or oligomenorrhea after CTX with cyclophosphamide. The ORM age (≥32 years) analyzed in series with AMH or AFC decrease the sensitivity in order to increases the specificity, making the result clinically useful with sensitivity and specificity around 80 % to amenorrhea or oligomenorrhea after CTX. Parallel analysis offered a higher sensitivity but lower specificity, as false-positives were more common. Serial analysis demonstrated lower sensitivity than the parallel analysis, resulting in a better performance when clinically employed; however, the specificity was higher. Serial analysis of age and AMH or AFC was demonstrated to be better than analysis of age separately. These parameters should be considered during pretreatment fertility preservation counseling.

## Methods

A cohort study was conducted. The study followed 52 eumenorrheic women younger than 40 years of age with breast cancer requiring CTX containing cyclophosphamide, who had not undergone previous CTX treatment. Patients were selected from six hospitals in Porto Alegre, Rio Grande do Sul, Brazil. The study protocol was approved by the corresponding Research Ethics Committees (HCPA #07-061) and was conducted in accordance with Brazilian guidelines and standards for human subject research (National Research Council Resolution 196/96).

Sample size was calculated based on the study by van Rooij [12]. The minimum number of 44 patients was estimated to find a difference of 1.4 ng/mL in AMH values between baseline and 6 months after completion of CTX with a significance level of 0.05 and a power of 90 %.

Patients were assessed through interview, blood sampling and ultrasound scan before and 6 months after CTX. The blood samples were centrifuged at 3500 rpm for 15 min and the serum was stored at −80 °C. Measurement of FSH and estradiol through chemiluminescence was performed using the ADVIA Centaur® XP Immunoassay System (Siemens®) (samples were collected at any time of menstrual cycle in order to avoid delay of chemotherapy begin). FSH and estradiol were measured on the day the patient performed transvaginal ultrasound, together with the other exams. It was not possible to wait for the best moment to collect this exams, since it could delay the onset of chemotherapy. AMH was measured through ELISA (Beckman Coulter, Genese Imunotech®, France), as described in the literature [23].

Patients were referred and the same researcher performed ultrasound scans in all patients with Siemens Sonoline Adara, an ultrasound device with vaginal probe of 5 MHz, any day within a menstrual cycle because of the urgency to begin CTX. All Follicles with a mean diameter between 2 and 10 mm were considered for AFC [24].

The analysis was performed with Statistical Package for Social Sciences (SPSS) 18. Results are presented as median and interquartile range (25–75 %) because the data of this study do not show Gaussian normal distribution. The data were tested with the Mann–Whitney test and the multiple comparisons were corrected through Bonferroni. The categorical variables were analyzed through Pearson Chi-Square test. A logistic regression for independent samples and determination of the ROC curve were performed. The significance level was considered *p* <0.05.

## Consent

Written informed consent was obtained from the patiens for the publication of this report and accompanying images.
